# Individual vs. Combined Short-Term Effects of Soil Pollutants on Colony Founding in a Common Ant Species

**DOI:** 10.3389/finsc.2021.761881

**Published:** 2021-10-29

**Authors:** Dimitri Seidenath, Anja Holzinger, Klara Kemnitz, Nico Langhof, Darleen Lücker, Thorsten Opel, Oliver Otti, Heike Feldhaar

**Affiliations:** ^1^Animal Population Ecology, Animal Ecology I, Bayreuth Center of Ecology and Environmental Research (BayCEER), University of Bayreuth, Bayreuth, Germany; ^2^Department of Ceramic Materials Engineering, University of Bayreuth, Bayreuth, Germany

**Keywords:** multiple stressors, soil pollution, insect decline, claustral colony founding, particulate matter, microplastic

## Abstract

Insects are integral to terrestrial life and provide essential ecosystem functions such as pollination and nutrient cycling. Due to massive declines in insect biomass, abundance, or species richness in recent years, the focus has turned to find their causes. Anthropogenic pollution is among the main drivers of insect declines. Research addressing the effects of pollutants concentrates on aquatic insects and pollinators, despite the apparent risk of contaminated soils. Pollutants accumulating in the soil might pose a significant threat because concentrations tend to be high and different pollutants are present simultaneously. Here, we exposed queens of the black garden ant *Lasius niger* at the colony founding stage to different concentrations and combinations of pollutants (brake dust, soot, microplastic particles and fibers, manure) to determine dose-dependent effects and interactions between stressors. As proxies for colony founding success, we measured queen survival, the development time of the different life stages, the brood weight, and the number of offspring. Over the course of the experiment queen mortality was very low and similar across treatments. Only high manure concentrations affected the colony founding success. Eggs from queens exposed to high manure concentrations took longer to hatch, which resulted in a delayed emergence of workers. Also, fewer pupae and workers were raised by those queens. Brake dust, soot and plastic particles did not visibly affect colony founding success, neither as single nor as multiple stressors. The application of manure, however, affected colony founding in *L. niger* negatively underlining the issue of excessive manure application to our environment. Even though anthropogenic soil pollutants seem to have little short-term effects on ant colony founding, studies will have to elucidate potential long-term effects as a colony grows.

## Introduction

The loss of biodiversity worldwide poses one of the biggest threats to ecosystem functioning and consequently to human well-being in the Twenty-first century (1–3). Human-induced vertebrate declines and species extinctions are well-documented and have long captured the attention of scientists and the broader public (4). Due to an increasing number of studies showing massive declines in insect biomass, abundance, or species richness over the last decades [(3, 5–7), but see: (8, 9)], the focus has recently turned to understanding the mechanisms behind insect declines.

Insects are an integral part of terrestrial and aquatic food webs as consumers and by linking primary producers with consumers of higher trophic levels. They provide many essential ecosystem functions such as pollination, regulation of herbivores and plants, or nutrient cycling through the decomposition of leaf litter and dead wood, or removal of dung (10). Consequently, further losses in insect diversity and biomass will result in a highly uncertain development of ecological processes potentially affecting human living as we know it.

One of the main drivers of insect declines, besides habitat conversion, land-use intensification, climate change, and biological factors, is anthropogenic pollution (7, 11, 12). Pollutants can originate from traffic, industrial production and agriculture, including pesticides (13). They enter the environment via deliberate application or leakage and poor waste management (14). Because agricultural intensification is one of the most apparent reasons for the observed declines in insects, research has strongly focused on the effects of pesticides and fertilizer application on insect health and fitness (12). However, especially in or close to urban areas, industrial pollution from heavy metals, airborne particulate matter, or plastic waste may also adversely affect insect populations (15, 16). A potential sink for such pollutants is soil, as contaminants can accumulate therein over centuries (13, 17). As a result, soils may contain mixtures of pollutants originating from various anthropogenic activities over the years. For soil-dwelling insects, such as ants nesting in soil, springtails, or beetles, this could be very problematic (18, 19).

Despite the apparent risk of contaminated soils, research addressing the effects of pollutants on insects concentrates on aquatic insects and pollinators (12). The focus on pollinators is evident, not only due to their ecological and economic value but also because many of them are eusocial insects. Social insects may be especially threatened by pollution for several reasons. They often have large foraging areas and transport food to their nest as a central storage place. The storage of food also likely accumulates pollutants in the nest. Such an accumulation may lead to chronic exposure to a mixture of pollutants to adults and their offspring (16). For example, in different compartments of honeybee hives, heavy metals and pesticides have been identified and shown to have negative effects on individual bees and colony development, primarily affecting brood stages negatively (20–24).

The effect of multiple stressors on insects has recently come into focus, and experiments with two or more stressors gain momentum (25, 26). Currently, many studies focus on the interplay of two or more pesticides (27, 28) or the interplay of pesticides with another stressor, such as climate change (29, 30), or pathogens (31–33). Many insects will likely be confronted with multiple-stressor scenarios under natural conditions in human-altered landscapes. The outcomes of these studies show different interactive effects. Some stressors interact antagonistically such as temperature diminishing adverse effects of pesticides (29, 34). Other studies found evidence for synergistic effects because exposure to multiple stressors increased negative effects in a non-additive way [overview in (16, 35)]. However, we still largely lack data investigating potential interactions between stressors and the form of the interaction in soil insects. Research in aquatic environments and in bees show that non-additive effects are quite common (36, 37). Understanding these interactive effects between multiple stressors in insects is vital when trying to unravel the complexity of insect declines and to predict how a combination of particular stressors will impact insects.

Ants are a prominent insect group in most terrestrial ecosystems with regard to species diversity and biomass (38). They are important ecosystem engineers due to their functions in soil perturbation, nutrient cycling, seed dispersal and as pest controllers (39–42). Even though many reports on the decline of Hymenoptera such as wild bees exist, the evidence for ant species declines is still sparse (12, 43). Increasing land-use intensity in temperate grasslands has been shown to result in a decrease in ant species richness and abundance (44–46). The drivers of this decrease were a higher frequency of mowing or fertilization (45). In agricultural land or other strongly human-impacted habitats, such as urban parks, roadside habitats or surroundings of industrial sites, ant species richness and abundance decrease [(47), overview in (48)]. Here, habitat fragmentation, habitat loss and soil pollution may drive the decline of ant diversity and abundance (47–50). In addition, neonicotinoid insecticides that are widely used in agriculture have been shown to lower colony growth-rate of ants (51).

As long-lived organisms and central place foragers, ants can be negatively affected by pollutants in their environment. Pollutants may accumulate in their bodies (49, 52) with adverse effects on individual and thus colony-level fitness [(49), overview in (16, 51, 53)]. Some ants, such as the black garden ant *Lasius niger*, live in a variety of different habitats, including agricultural and urban areas (54). The soil in such areas may be contaminated with a mixture of anthropogenic pollutants, such as microplastic deriving from degradation of larger plastic litter, airborne particulate matter from traffic and industrial processes such as brake dust or soot, or manure as fertilizer that is commonly applied to arable land and grasslands. The number of microplastic particles in the soil varies widely between sites, with concentrations of up to 6.7% (w/w) in industrial areas (55, 56). Identifying and quantifying airborne particulate matter in soil is complicated as the elemental composition may overlap with natural soil components. However, unnaturally high amounts of metals can be attributed to external sources such as brake dust (57, 58). Isotopic analyses revealed up to 0.54% (w/w) of urban soils in Arizona as soot carbon black (59). As for manure, the European law allows application of up to 35 tons per year per hectare, resulting in large quantities on agricultural fields and grasslands (60).

In many ant species, like *Lasius niger*, queens found new colonies during a claustral phase. They build a nest in the soil and raise their first brood by metabolizing stored body reserves by histolysis of their flight muscles (38, 61). Only a minority of young queens successfully manages to found a colony. Predators catch many queens during their nuptial flight and subsequent search for a suitable nest site (38, 62). Nesting in soil, queens potentially encounter many pollutants during the claustral phase of colony founding, affecting the queen's fitness and the development of their brood. Negative effects of pollutants in the soil may therefore further diminish the ratio of queens successfully founding a colony.

To test this hypothesis, we exposed *Lasius niger* queens at the claustral colony founding stage to five pollutants in two concentrations to reveal the potential effects of different soil pollutants on ants. We simulated different soil contaminations by mixing soil with brake dust, soot, microplastic particles, microplastic fibers, and manure. We compared the effects of each pollutant alone to multiple stressor environments with combined pollutants. Finally, we measured the development time of the different life stages and queen survival. Once workers were present, we measured the brood weight and the number of offspring as a proxy for colony founding success. Except for water, the queens do not take up any food during claustral colony foundation. Therefore, we do expect marginal lethal effects of the pollutants on the queens themselves, as toxic effects would mostly be exerted via contact of the cuticle with the contaminated soil. However, since the brood and especially larvae have a thinner cuticle than the queens, these life stages may be negatively affected directly when in contact with contaminated soil. The presence of pollutants could cause stress in the queens, leading to reduced investment in the brood due to allocation costs (63), since pollutants may be taken up by the queen when cleaning the surface of larvae that have come into contact with the contaminated soil or feeding during brood care.

Moreover, pollutants may alter the microbial community in the soil, which could affect the founding process (64). Even though we expect to find negative effects of single pollutants, it might well be that significant effects only manifest when ant queens or brood are exposed to a combination of pollutants. Organisms might be able to compensate for single effects but will be overstrained when facing multiple stressors.

## Materials and Methods

### Ant Queen Collection and Housing

Between 9th and 12th July 2020 we collected 600 *L. niger* wingless queens after their nuptial flight in and around Bayreuth (Bavaria, Germany). We kept them in plastic boxes containing damp paper towels until further use. On 13th July, 510 queens were randomly assigned to 16 soil treatments and one control treatment (*N* = 30 queens per treatment) using Research Randomizer (65). For this, we prepared 15 ml falcon tubes as nests for the queens with the different soils. Each tube was filled with 5 ml autoclaved water and a cotton ball pushed to the 5.5 ml mark to provide constant moisture. On top of the cotton ball, we put 5 ml of the respective soil treatment. The soils contained different types, concentrations and combinations of pollutants (For details see “Preparation of Soil Treatments”). Finally, we placed one queen into each tube, closed the screw-top loosely to ensure air circulation and then started the experiment.

During the experiment, the queens were kept in a climate chamber at 20°C and 70% humidity under a constant 12:12 h light:dark cycle. Only the regular checks for queen survival, the presence of the brood stages and burrowing depth were performed in the laboratory at room temperature. Brood stages included eggs, larvae, pupae and freshly hatched workers. We checked tubes every other day until eggs or the next brood stage appeared. Then, we checked daily for a whole week. For the checks, we used a dissecting microscope to identify brood stages clearly. Observers were blind regarding the treatment. After the first worker hatched, the tube, including the queen and the brood, was frozen at −20°C until further assessment to compare colony founding success at a defined stage. In cases where no workers emerged, queens were frozen 60 days after the start of the experiment. Finally, we sorted, counted, and weighed each queen and its brood (dry weight after 48 h at 50°C). For each queen, we calculated the development times of brood stages by deducting the days of the first emergence from each other. As we froze the brood when the first worker appeared, worker count alone is not a meaningful variable as sometimes more than one worker hatched at the same time by chance. Consequently, for brood count, we add up the number of pupae and workers to have only one variable.

### Preparation of Soil Treatments

The soil used in our experiment was provided by the Ecological Botanical Garden of the University of Bayreuth and consisted of low-nutrient cultivation soil mixed with 10% organic compost. Before mixing it with pollutants the soil was dried for overnight at 70°C in a drying oven (UFE 600, Memmert, Schwabach, Germany) and sieved. We added autoclaved water (20% v/v) to the soil to establish the same moisture in each soil treatment. Then we used the following pollutants to prepare the soil treatments: brake dust particles, soot particles, polystyrene particles, polystyrene fibers and liquid manure.

#### Brake Dust Particles

Brake dust particles were provided by the Department of Ceramic Materials Engineering of the University of Bayreuth. Tribologically tested LowMet brake pads (provided by TMD company) were ground, after several braking cycles on a ceramic brake disc, that means after a dissipation of a total friction energy of about 15 MJ and temperatures up to 400°C. In order to reach the required fine-grained powder, 3 min in total, a vibrating cup mill with tungsten carbide grinding set up (pulverisette 9, Fritsch GmbH, Idar-Oberstein, Germany) was applied. A breakdown of the composition of such brake pads can be found in Breuer and Bill (66). The biggest fractions consist of steel wool [15% (w/w)], petrol coke [12% (w/w)], sulfides [10% (w/w)] as well as aluminum oxide and binder [both 5% (w/w)] (66). The particle sizes of the ground brake pads were measured using a laser diffraction particle size analyzer (PSA 1190 LD, Anton Paar GmbH, Ostfildern-Scharnhausen, Germany). The average particle size found was 10.19 ± 4.37 μm (D10 = 0.68 μm, D50 = 5.76 μm, D90 = 25.87 μm).

#### Soot Particles

We used the carbon black PRINTEX 30 Furnace Black (Degussa AG, Frankfurt, Germany) for the soot treatments with an average primary particle size of 27 nm. Carbon black and soot are often used interchangeably even though they are distinct from each other. Carbon blacks are commercially produced elemental carbon particles with different properties (67). In contrast, soot is a by-product of relatively uncontrolled, incomplete combustions, which results in a material of varying and often unknown composition (68). In terms of particle size, there is a high degree of similarity between soot and carbon blacks (69). As we want to simulate contaminated soil and since soot is the most similar, naturally occurring pendant we henceforth refer to the carbon black as soot.

#### Polystyrene Particles and Fibers

Granules were ordered from Styrolution (Frankfurt am Main, Germany) and further processed to particles and fibers by the faculty of Macromolecular Chemistry I (MCI) at the University of Bayreuth. Polystyrene particles had a particle size of 125–200 μm, while the fibers had a length of 1–4 mm and a diameter of 40 μm.

#### Liquid Manure

Liquid manure was provided by a small dairy cattle farm in Bauerngruen near Bayreuth on 10th July 2020 (49.894071, 11.587649). The liquid manure was collected directly from the outlet of the stable for calves (where ~20 calves are kept at a time) that do not receive any treatment with antibiotics according to the farmer. However, it is likely that the calves have received deworming treatment. As manure is typically applied in liquid form, we did not dry the manure. Also, drying the manure likely changes its properties making it less comparable to the common practice in agriculture.

**Figure 1:**
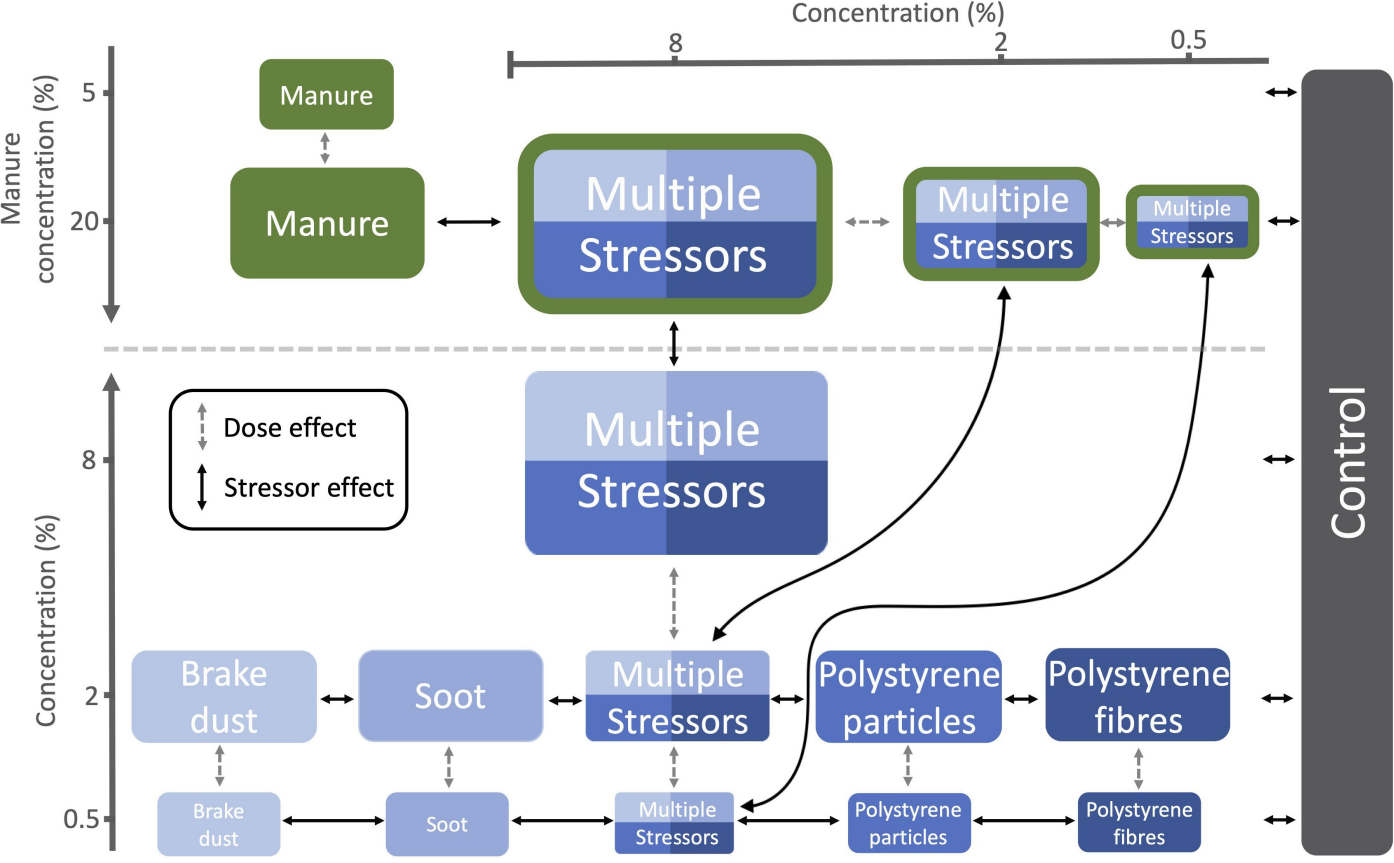
The different treatments and their stressor concentrations (v/v). Relevant comparisons are comprised between treatments that lie on a vertical or horizontal line or are connected with arrows. Dashed arrows represent the dose effect, i.e., the comparisons within stressor type. Solid arrows represent the stressor effect, i.e., the comparisons between stressors types or stressor type and control.

We assessed individual and dose-dependent effects of each solid pollutant by using single pollutant treatments with two different concentrations [0.5 and 2% (v/v)] of each pollutant mixed into the soil. In the liquid manure treatments, we replaced the water added to the soil completely [20% (v/v)] or partly with liquid manure [5% (v/v)]. In the multiple stressor treatments, we mixed the four solid pollutants in equal proportions with three different overall concentrations [0.5, 2, and 8% (v/v)] into the soil, either with or without manure [20% (v/v)], to see combinatorial effects. We chose the concentrations to assess stressor effects and dose effects (For details see: “Statistical data analyses”; Figure 1). Because studies have found a wide range of pollutant concentrations in natural soils [up to 6.7% (w/w)] (56), we chose a similar range for our experiment. However, for conceptual consistency we used volume/volume concentrations, as particles differ dramatically in their bulk density.

### Statistical Data Analyses

All statistical analyses were performed using R 4.0.3 (70). We excluded queens that were untraceable after some time (4.1%), mostly due to burrowing in the soil, and were not observed again until freezing from the survival analysis. The survival data were analyzed with a Cox proportional hazard regression (COXPH) with treatment as a predictor [package *survival*, (71, 72)]. Survival model assumptions were tested using Schoenfeld residuals [*survminer*, (73)].

For all subsequent analyses, we excluded queens that died during the experiment (*N* = 36), were untraceable for at least 10 days (*N* = 21) or had zero or more than five workers at the point of freezing (*N* = 35). More than five workers at the point of freezing indicated that we missed the day of first emergence because normally only few workers emerge within 1 day. Queen weight, brood weight, brood counts and development times were analyzed fitting generalized linear models GLMs with treatment as a predictor. We checked model assumptions using model diagnostic test plots, i.e., qqplot and residual vs. predicted plot from the package *DHARMa* (74). Depending on model assumptions, we then used Kruskal-Wallis tests or produced F-statistics with the function *anova()* to calculate *p*-values for differences between treatments. For significant treatment effects, we ran pairwise comparisons. In the case of a significant Kruskal-Wallis test, pairwise comparisons were done using Dunn's test for multiple comparisons with Benjamini-Hochberg correction [package “FSA,” (75)]. In the case of a significant ANOVA, pairwise comparisons were made using Tukey HSD *post-hoc* test with Benjamini-Hochberg correction from the package *multcomp* (76). Even though we ran all possible pairwise comparisons, we only report the relevant comparisons because comparing different stressor types with different concentrations is not very informative. To characterize the stressor effects, we show the comparisons of each stressor type to the control, the comparisons between stressor types of the same concentration and the comparisons between the high concentration of manure and the multiple stressor treatments with manure. Finally, we compared the treatment levels within the same stressor type to identify dose effects (Figure 1). To measure effect sizes of the pairwise-comparisons, we calculated Hedges' G with the package *esvis* (77). Data were arranged using the package *tidyr* (78) and were plotted using the package *ggplot2* (79).

## Results

Overall, we found very low mortality in the queens. Thirty-six of 510 queens (7.1%) died during the experiment. Queen survival was similar across treatments (COXPH overall LR-test: *X*^2^ = 23.23, *df* = 16, *P* = 0.108).

Queen weight significantly differed between the treatments [GLM with gamma distribution: *F*_(16, 324)_ = 1.896, *P* = 0.020]. However, only queens exposed to the high concentration of manure were significantly heavier than the queens exposed to the low concentration of manure (Table 1, Supplementary Figure 1, Tukey comparison: *P* = 0.044). Even though treatment significantly affected brood weight [GLM with gaussian distribution, *F*_(16, 324)_ = 2.168, *P* = 0.006], the multiple comparison analyses showed no significant differences between the treatment levels (Table 1, Supplementary Figure 2). At higher manure concentrations brood weight tended to be lower than the control. But for the other pollutants we could not identify a clear pattern.

**Table 1:** *P*-values and Hedges' g of the relevant comparisons with a *p*-value below 0.1 for the different response variables.

**Response**	**Comparison**	**Mean difference**	***p*-value**	**Hedges' g**
**Weight (mg)**
Queen	Manure (20%) > Manure (5%)	1.524	**0.044**	1.123
Brood	Control > Manure (20%)	1.019	0.055	0.964
	Control > Multiple stressors (2%) + Manure (20%)	1.133	0.052	0.937
	Control > Multiple stressors (0.5%) + Manure (20%)	0.943	0.092	0.916
	Multiple stressors (0.5%) > Multiple stressors (0.5%) + Manure (20%)	0.908	0.092	0.938
**Number of**
Eggs	–	–	–	–
Larvae	–	–	–	–
Pupae + worker	Control > Manure (20%)	4.146	0.068	0.906
	Control > Multiple stressors (0.5%) + Manure (20%)	4.313	0.068	1.005
	Control > Multiple stressors (2%) + Manure (20%)	5.174	**0.030**	0.901
	Manure (5%) > Manure (20%)	3.662	0.080	0.747
	Multiple stressors (0.5%) > Multiple stressors (0.5%) + Manure (20%)	5.950	**0.004**	1.408
	Multiple stressors (2%) > Multiple stressors (2%) + Manure (20%)	4.389	0.064	0.753
**Development time (days)**
Egg to larvae	Manure (20%) > Control	1.521	**0.002**	0.893
	Manure (20%) > Manure (5%)	2.018	**<0.001**	1.298
	Multiple stressors (0.5%) + Manure (20%) > Control	2.188	**0.009**	0.944
	Multiple stressors (0.5%) + Manure (20%) > Multiple stressors (0.5%)	2.550	**<0.001**	1.207
	Multiple stressors (2%) + Manure (20%) > Control	1.632	**0.026**	0.834
	Multiple stressors (2%) + Manure (20%) > Multiple stressors (2%)	2.278	**<0.001**	1.426
	Multiple stressors (8%) + Manure (20%) > Control	0.952	0.051	0.620
	Multiple stressors (8%) + Manure (20%) > Multiple stressors (8%)	1.492	**0.002**	1.453
Larvae to pupae	Manure (20 %) > Control	0.854	0.068	0.783
	Multiple stressors (0.5 %) + Manure (20 %) > Control	1.213	**0.050**	0.613
	Multiple stressors (8 %) + Manure (20 %) > Control	1.224	0.060	0.815
Pupae to worker	–	–	–	–
Egg to worker	Manure (20%) > Control	1.958	**0.010**	0.955
	Manure (20%) > Manure (5 %)	2.478	**<0.001**	1.265
	Multiple stressors (0.5%) + Manure (20%) > Control	2.725	**0.025**	0.998
	Multiple stressors (0.5%) + Manure (20%) > Multiple stressors (0.5%)	3.650	**<0.001**	1.519
	Multiple stressors (2%) + Manure (20%) > Control	1.708	0.087	0.724
	Multiple stressors (2%) + Manure (20%) > Multiple stressors (2%)	2.167	**0.008**	0.952
	Multiple stressors (8%) + Manure (20 %) > Control	1.493	0.064	0.748
	Multiple stressors (8%) + Manure (20%) > Multiple stressors (8%)	2.163	**0.004**	1.472

*According to Cohen (80) effect sizes > 0.8 indicate a large effect. Significant adjusted P-values are shown in bold*.

Neither the number of eggs [GLM with gaussian distribution: *F*_(16, 324)_ = 1.601, *P* = 0.067], nor the number of larvae differed between treatments [GLM with gaussian distribution: *F*_(16, 324)_ = 1.668, *P* = 0.051; Supplementary Figures 3, 4]. In contrast, the number of pupae and workers significantly differed between treatments [GLM with gaussian distribution: *F*_(16, 324)_ = 2.852, *P* < 0.001]. Queens exposed to the high concentration of manure had fewer pupae and workers than control queens [Tukey comparison control vs. multiple stressors (2%) + manure: *P* = 0.030] and queens exposed to multiple stressors [Tukey comparison multiple stressors (0.5%) vs. multiple stressors (0.5%) + manure: *P* = 0.004; Table 1, Supplementary Figure 5].

**Figure 2:**
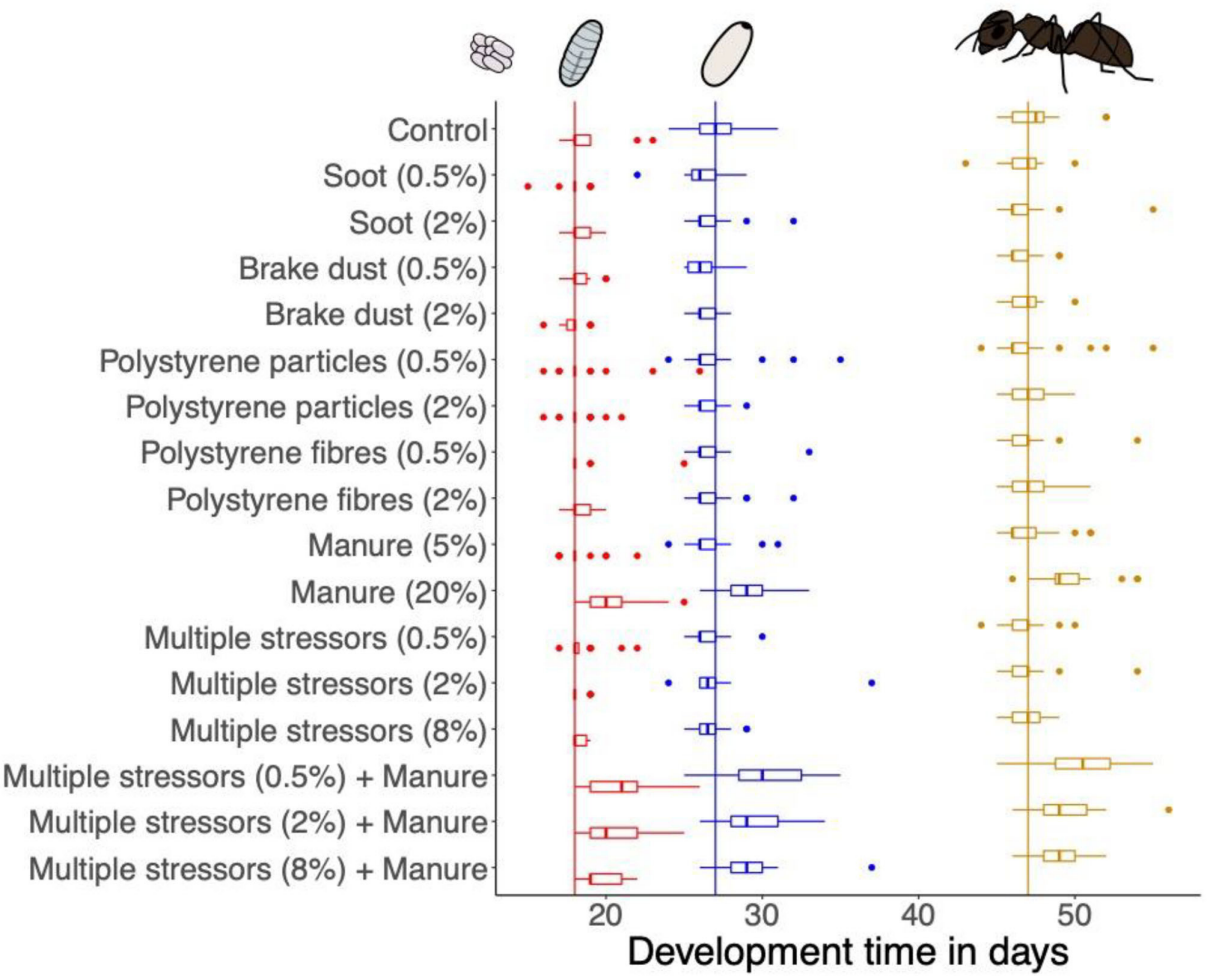
Development time of the brood stages of each treatment (days since first appearance of eggs). Boxplots show median, first, and third quartile. Dots show outliers outside of 1.5 × Inter-quartile range. Horizontal lines indicate the median of each brood stage over all treatments (red = larvae, blue = pupae, yellow = worker).

The development time from egg to larvae significantly differed between treatments (Kruskal-Wallis rank sum test: *X*^2^ = 112.86, *df* = 16, *P* < 0.001). The development time from egg to larvae was longer in treatments containing the high concentration of manure compared to controls (Figure 2, Dunn's comparisons: see Table 1). We also found a significant effect of treatment on the development time from larvae to pupae (Kruskal-Wallis rank sum test, *X*^2^ = 39.311, *df* = 16, *P* < 0.001). However, *post-hoc* Dunn's test revealed no significant differences between the treatments (Table 1, Figure 2). The development time from pupae to worker did not differ between treatments (Kruskal-Wallis rank sum test, *X*^2^ = 14.417, *df* = 16, *P* = 0.568; Figure 2). The overall development time from egg to worker differed among treatments (Kruskal-Wallis rank sum test, *X*^2^ = 88.944, *df* = 16, *P* < 0.001). Similar to the development time from egg to larvae, the development time from egg to worker was longer in the treatments with the high concentration of manure compared to controls and multiple stressor treatments (Figure 2, Dunn's comparisons: see Table 1).

## Discussion

In this study, we looked at the effects of different soil pollutants on the colony founding success of *Lasius niger* ant queens in the laboratory. Ants were exposed to different concentrations and combinations of pollutants to determine dose-dependent effects and interactions between these potential stressors. While brake dust particles, soot and polystyrene microplastic (particles or fibers) did not affect any of the measured colony foundation parameters, a high concentration of manure in the soil led to delayed egg development and a smaller number of pupae and workers.

The overall ant queen mortality was very low (7.1%) and was not affected by soil treatment. This indicates that pollutants in the soil, at least those used here, do not exert toxic effects on the ant queens. During colony founding, ant queens do not consume any food as they meet their energy demands by using internal resources, such as degeneration of their flight muscles (38, 61). For a pollutant to be toxic at this stage, it would have to be lethal at a very low dose or capable of entering via the cuticle or the trachea. Consequently, most pollutants at field-realistic doses, such as insecticides and fungicides, do not increase mortality in founding ant queens [(51, 81, 82)), but see (83)].

While we found no differences in queen survival, the number of offspring or brood weight, exposure to a high concentration of manure (20% v/v) delayed brood development time resulting in a delayed time of first worker emergence. A slower development from egg to larvae caused this delay (Figure 2). While we are not aware of any study investigating the effects of manure on the development of soil-dwelling insects, the effects on soil properties, such as pH or oxygen, or on microbial soil communities are well-studied. Even though we did not measure it, manure typically changes soil pH and increases the availability of nutrients, which in turn increases microbial biomass (84, 85). This shift in pH and biomass affects the structure of the microbial soil community (86–88). Such changes in the soil may also affect the development of larger organisms. Because ant development varies with environmental conditions (89), the delayed egg development may be explained by manure-induced changes in oxygen-levels. As insect eggs depend on oxygen for their development, they have diffusion holes in the shell (90, 91). Under low oxygen levels in the immediate environment, *Tenebrio molitor* (92) and *Drosphila melanogaster* (93) show slower development. The application of manure reduces oxygen levels in the soil, sometimes locally even leading to anoxic areas (94, 95). Peak oxygen deficits in soil occur 16 h after the manure application and go back to near-normal within the following days (94). We assume this process could explain the observed delay in the development time of the brood stages in our study. Because an oxygen deficit only manifests for a short time after the manure was added, it most likely just affected the early brood development. As most queens (94%) laid their eggs within the first 2 days, delayed egg development could have been caused by low oxygen levels. Later brood stages were not affected since oxygen should have been back to normal after a few days. However, as we did not measure oxygen levels during our study, this explanation remains hypothetical. The smaller number of pupae and workers in the manure treatments might be explained in a similar way. Low oxygen levels at the beginning of the experiments may have not only caused delayed development but also lead to losses in the first egg clutch (91). Those early losses would result in fewer pupae and workers at the time of first worker emergence. A smaller number of workers and a delayed development could lead to less competitive ant colonies. As ants are in constant competition for resources and habitat, a smaller colony size at an early stage may reduce the survival probability of affected colonies (96). Repeated treatment of grasslands with manure can then add up to the observed slight but significant negative effects of fertilization on ant species richness and abundance (45).

The manure we used for our experiment was collected directly from the sewage of a stable that houses calves that were not treated with antibiotics. However, large-scale cattle farming typically relies on high amounts of antibiotics and dewormers (97, 98). The effect of manure that contains residues of medications could be different from the effects we observed as it is likely that that soil fauna is even more affected (99). Before application, manure is often aged in a lagoon in which bacteria degrade organic matter (100). Again, such manure could have different effects than the ones we observed, as many of the organic compounds are already decomposed (101). Because we used nearly sterile soil (dried overnight at 70°C + use of autoclaved water) in our setup, the effects of manure in our experiment could partly be attributed to microorganisms. Manure typically carries high loads of different microorganisms. Therefore, the manure treatments also represent a bacteria-rich environment, at least in comparison to our other almost sterile soil treatments. Ant queens maintain their own and their nest's hygiene by investing in external immunity, which can come at the cost of reproduction (64, 102, 103). *L. niger* queens founding a colony under high microbial pressure are actually forced to pay a substantial cost by simultaneously investing in reproduction and immunity (64). Therefore, the negative impact of manure in our experiment could also partly be caused by the similar effect of microbes on colony development described by Tragust et al. (64).

Apart from manure none of the other pollutants applied, i.e., brake dust, soot, microplastic particles and fibers, caused any changes in the investigated colony founding parameters. It is highly unlikely that initial queen weight affected this finding as we have fully randomized the assignment of the queens to the different treatments using an automated algorithm (65). Nevertheless, it would be interesting to investigate a potential effect of pollutants on the relation between initial queen weight and colony founding success. Although other studies show that fecundity and brood development of ants are sensitive to sublethal concentrations of pollutants, we did not detect any effects on those parameters in *L. niger*. In contrast, azole fungicides decrease fecundity in *L. niger* and the semi-claustral founding ant *M. rubra* (81, 83, 104). Thiamethoxam, a neonicotinoid insecticide, negatively impacted *L. niger* resulting in smaller colony size and worker weight (51). Selenium, a widespread contaminant in soils resulting from agricultural irrigation, hindered brood production in the Argentine ant *Linepithema humile* (105). A field experiment revealed negative effects of microplastic pollution on soil fauna, including ants (106). However, studies investigating the direct effects of microplastic on ants or studies that try to detect microplastic in ant bodies are still lacking. The combination of pollutants in the multiple stressor treatments revealed no additional effects other than those induced by treatments with a high concentration of manure. Therefore, we found no notable interactions among the different pollutants that would cause different effects on the ant queens.

Even though we did not find any effects of the pollutants apart from manure during our experiment, we cannot yet declare the pollutants as harmless to founding *L. niger* queens, as our experimental setup had some limitations. The commercially produced carbon black we used as soot is chemically distinct from real soot. Soot has a higher proportion of organic compounds which might have affected the ants differently (67). Similar, the brake dust in our experiment was artificially produced by grounding brake pads. Brake dust from real braking processes may differ in chemical and physical composition and thus affect ants differently. Another limitation of our study is that the period until the emergence of the first worker may not be sufficient to detect mid- or long-term effects of pollutant exposure. Several studies show that ants can compensate for stress for some time but ultimately must pay the hidden costs later in life. Some effects of a fungal pathogen in combination with physical stress on claustral colony founding ant *Crematogaster scutellaris* were only present at an early stage. In contrast, others only became evident in the long-term (107). The impact of microbe-enriched soil on *L. niger* queens just appeared when they were forced to lose their first batch of brood after hibernation (64). Another study found no effects of thiamethoxam on *L. niger* colonies before the first overwintering, but exposed colonies had fewer workers and larvae before the second winter (51).

Apart from long-term effects, other hidden costs might be present that our study design cannot uncover. The pollutants could affect worker health and immunity. Eg., heavy metal pollution suppressed the encapsulation response in wild colonies of *Formica aquilonia* (53). Ant workers may be more prone to the pollutants than the queen, as a recent study suggests a superior detoxification system in ant queens (51). In honeybees (*Apis mellifera*), queens are much more tolerant to acaricides than workers, even when adjusted for body size (108).

The black garden ant *L. niger*, a prevalent ant species across Europe, has a wide range of habitats, including urban areas and agricultural fields (54). The frequent occurrence might be explained by a higher stress tolerance of *L. niger* than other species. Higher resilience to disturbance and pollutants forms an important trait to tolerate and survive in human-altered landscapes. Genomic analysis revealed an increased potential of stress-resistance in *L. niger* compared to other ant species (109). The higher number of cytochrome P450 genes present in *L. niger* could improve its detoxification abilities of anthropogenic pollutants. Moreover, *L. niger* prefers visual information over pheromone trails for foraging, making it less vulnerable to interferences with repellent substances that could be especially present in urban environments (110, 111). These findings suggest a higher tolerance of *L. niger* against pollutants than other ants, even though we do not know of any study explicitly testing this hypothesis. Studies of *Formica* s. str. in heavy metal polluted areas showed that even closely related species can differ in their sensitivity to pollutants (49). Consequently, even though we do not find any short-term effects of pollutants on *L. niger*, we cannot conclude that there are no effects on ants in the long-term or that the pollutants studied here have more detrimental effects on other ant species. Especially rare ant species may be more vulnerable to pollution.

We could show that single and combined exposure of different soil pollutants does not affect colony founding in *L. niger* until the first workers emerge. The application of manure, however, affected colony founding by prolonging the development time from egg to larvae which ultimately led to a delayed emergence of the first workers. Moreover, fewer pupae and workers were raised by the queens in the manure treatments. These findings underline the issue of excessive manure application in our environment. Even though we did not find any effects or interactions among the other pollutants, effects on later stages of colony development cannot be ruled out. Therefore, future studies could investigate potentially hidden long-term effects of pollutants on colony development. Of similar importance might be to show if and how ant queens take up the pollutants.

## Data Availability Statement

The raw data supporting the conclusions of this article will be made available by the authors, without undue reservation.

## Author Contributions

DS, AH, OO, and HF conceived the idea, designed the experiment, and wrote the manuscript. AH, NL, and TO produced the particles. DS, KK, and DL carried out the experiment. DS, KK, and OO performed the statistical analysis. DS, AH, DL, KK, OO, and HF interpreted the results. All authors read and approved of the final manuscript.

## Funding

This project was funded by the Deutsche Forschungsgemeinschaft (DFG, German Research Foundation)–Project Number 391977956–SFB 1357 and by the Bavarian State Ministry of the Environment and Consumer Protection as part of the project network BayOekotox. This publication was funded by the University of Bayreuth Open Access Publishing Fund.

## Conflict of Interest

The authors declare that the research was conducted in the absence of any commercial or financial relationships that could be construed as a potential conflict of interest.

## Publisher's Note

All claims expressed in this article are solely those of the authors and do not necessarily represent those of their affiliated organizations, or those of the publisher, the editors and the reviewers. Any product that may be evaluated in this article, or claim that may be made by its manufacturer, is not guaranteed or endorsed by the publisher.

## Supplementary Material

Supplementary Figure 1 Queen weight of the different treatments. Boxplots show median, first and third quartile. Dots show outliers outside of 1.5 × Inter-quartile range.

Supplementary Figure 2 Brood weight of the different treatments. Boxplots show median, first and third quartile. Dots show outliers outside of 1.5 × Inter-quartile range.

Supplementary Figure 3 Number of eggs of the different treatments. Boxplots show median, first and third quartile. Dots show outliers outside of 1.5 × Inter-quartile range.

Supplementary Figure 4 Number of larvae of the different treatments. Boxplots show median, first and third quartile. Dots show outliers outside of 1.5 × Inter-quartile range.

Supplementary Figure 5 Number of pupae and workers of the different treatments. Boxplots show median, first and third quartile. Dots show outliers outside of 1.5 × Inter-quartile range.
