# Supplementary figures and images for: Individual vs. Combined Short-Term Effects of Soil Pollutants on Colony Founding in a Common Ant Species
Source: Front Insect Sci. 2021 Oct 29;1:761881. doi: 10.3389/finsc.2021.761881 (PMC10926528; doi:10.3389/finsc.2021.761881)

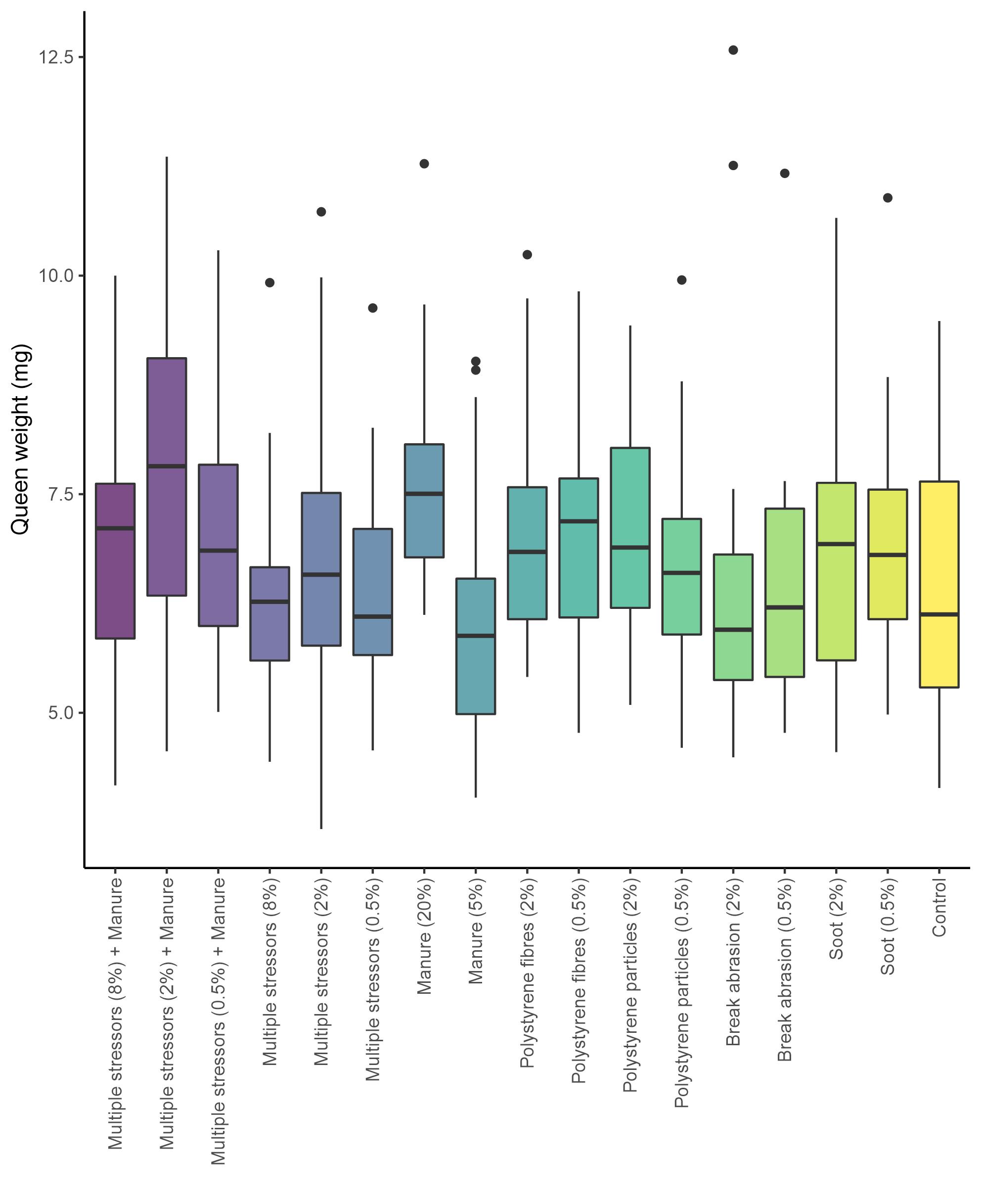

Supplement: Supplementary Figure 1 — Queen weight of the different treatments. Boxplots show median, first and third quartile. Dots show outliers outside of 1.5 × Inter-quartile range. [file Image_1.JPEG]

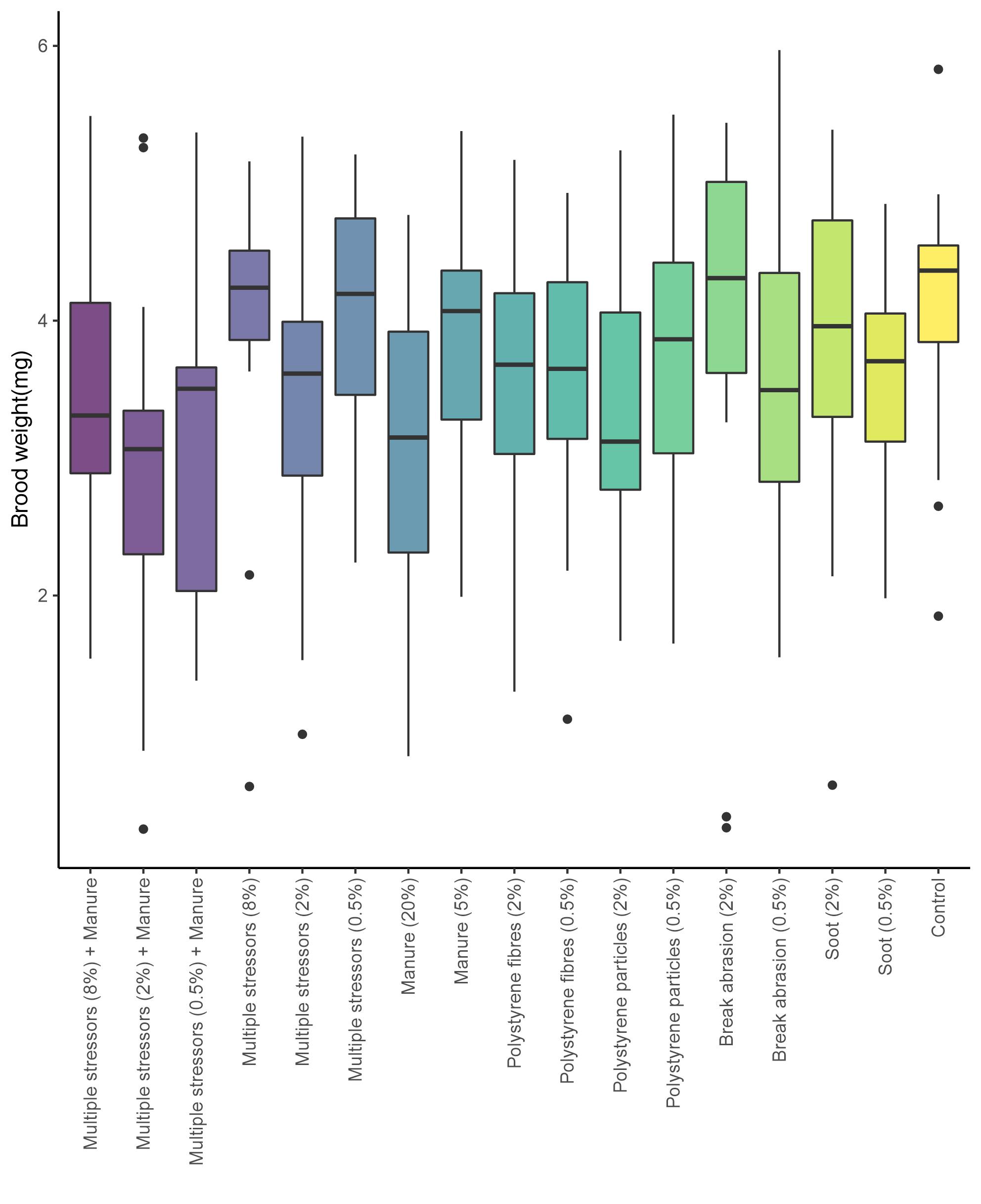

Supplement: Supplementary Figure 2 — Brood weight of the different treatments. Boxplots show median, first and third quartile. Dots show outliers outside of 1.5 × Inter-quartile range. [file Image_2.JPEG]

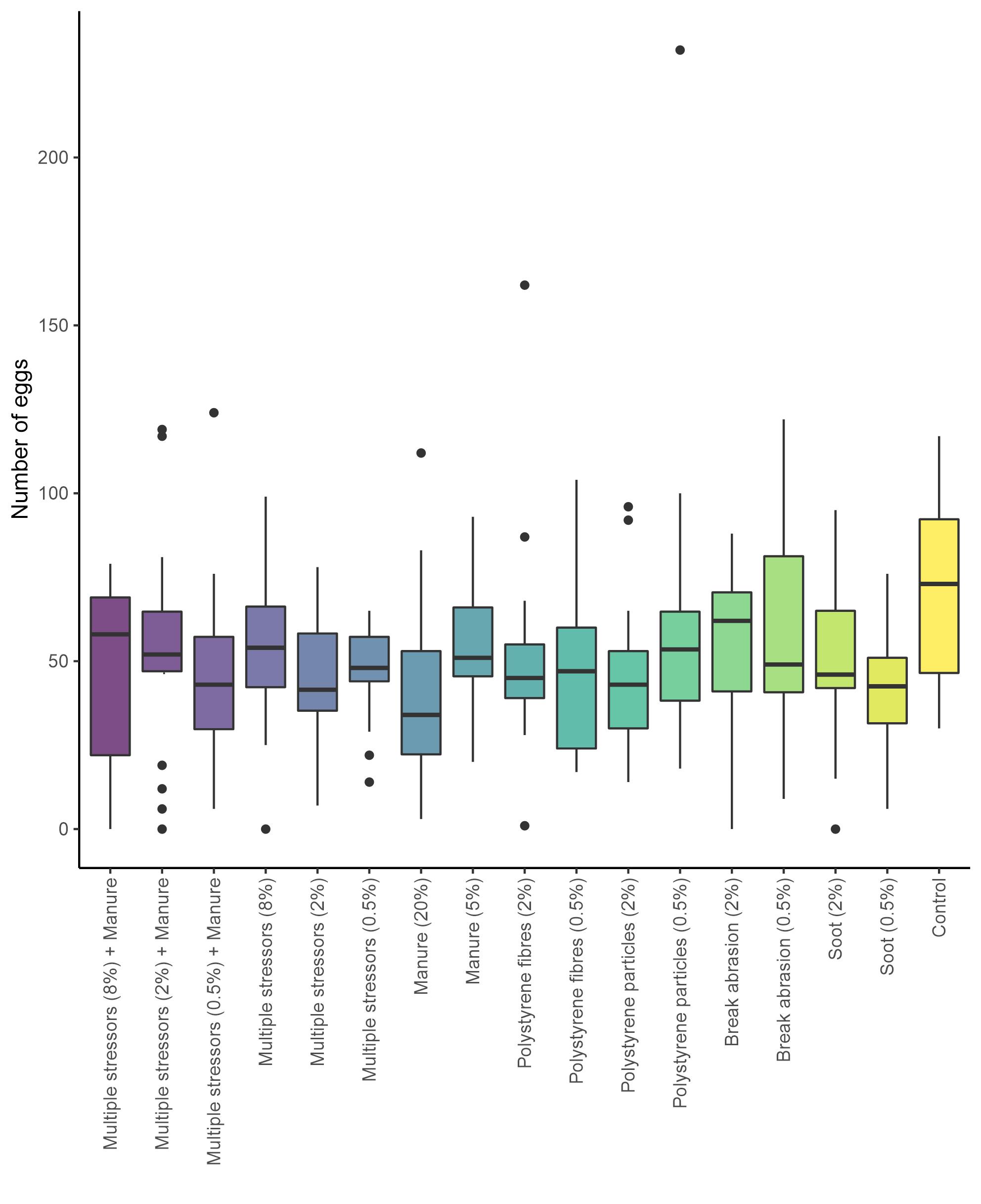

Supplement: Supplementary Figure 3 — Number of eggs of the different treatments. Boxplots show median, first and third quartile. Dots show outliers outside of 1.5 × Inter-quartile range. [file Image_3.JPEG]

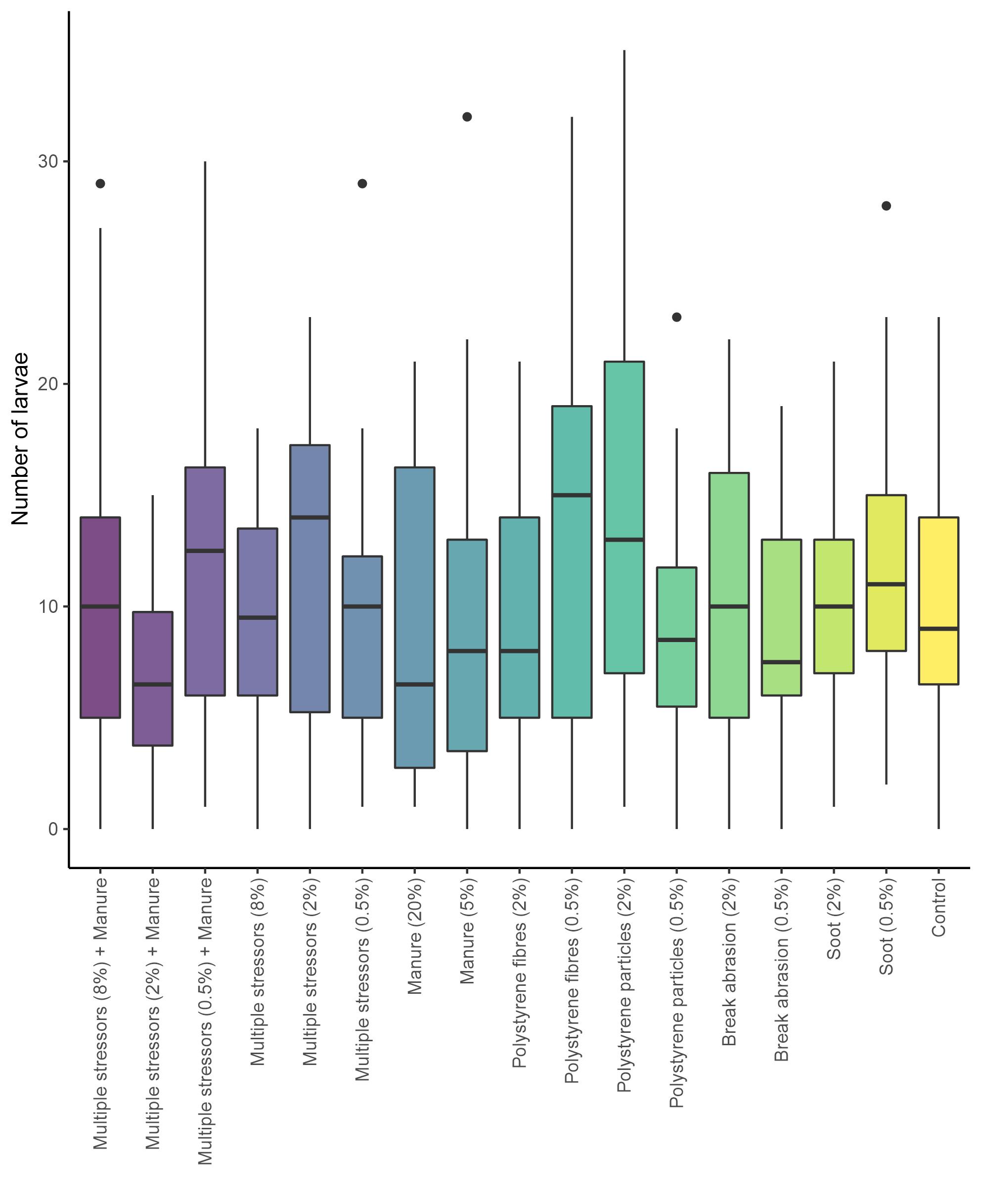

Supplement: Supplementary Figure 4 — Number of larvae of the different treatments. Boxplots show median, first and third quartile. Dots show outliers outside of 1.5 × Inter-quartile range. [file Image_4.JPEG]

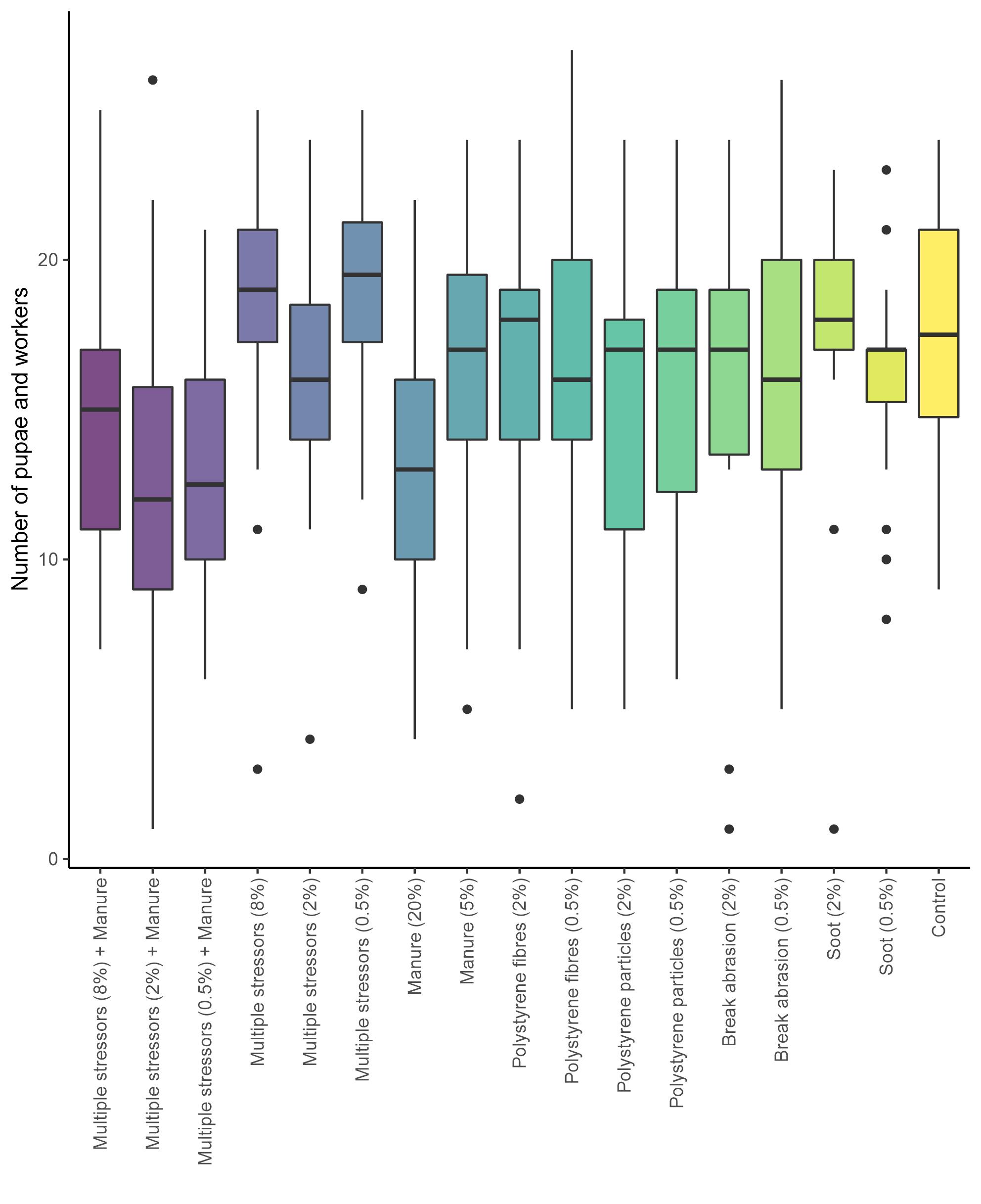

Supplement: Supplementary Figure 5 — Number of pupae and workers of the different treatments. Boxplots show median, first and third quartile. Dots show outliers outside of 1.5 × Inter-quartile range. [file Image_5.JPEG]
